# Direct Metal Forming of a Microdome Structure with a Glassy Carbon Mold for Enhanced Boiling Heat Transfer

**DOI:** 10.3390/mi9080376

**Published:** 2018-07-28

**Authors:** Jun Kim, Dongin Hong, Mohsin Ali Badshah, Xun Lu, Young Kyu Kim, Seok-min Kim

**Affiliations:** 1 Department of Mechanical System Engineering, Chung-Ang University, Heukseok-dong, Dongjak-gu, Seoul 06974, Korea; zuhn@cau.ac.kr (J.K.); hdi2305@naver.com (D.H.); 2 Department of Mechanical Engineering, Chung-Ang University, Heukseok-dong, Dongjak-gu, Seoul 06974, Korea; mohsinali@cau.ac.kr (M.A.B.); luxun@cau.ac.kr (X.L.); kykdes@cau.ac.kr (Y.K.K.)

**Keywords:** direct metal forming, glassy carbon micromold, enhanced boiling heat transfer, metallic microstructure

## Abstract

The application of microtechnology to traditional mechanical industries is limited owing to the lack of suitable micropatterning technology for durable materials including metal. In this research, a glassy carbon (GC) micromold was applied for the direct metal forming (DMF) of a microstructure on an aluminum (Al) substrate. The GC mold with microdome cavities was prepared by carbonization of a furan precursor, which was replicated from the thermal reflow photoresist master pattern. A microdome array with a diameter of 8.4 μm, a height of ~0.74 μm, and a pitch of 9.9 μm was successfully fabricated on an Al substrate by using DMF at a forming temperature of 645 °C and an applied pressure of 2 MPa. As a practical application of the proposed DMF process, the enhanced boiling heat transfer characteristics of the DMF microdome Al substrate were analyzed. The DMF microdome Al substrate showed 20.4 ± 2.6% higher critical heat flux and 34.1 ± 5.3% higher heat transfer coefficient than those of a bare Al substrate.

## 1. Introduction

Various research studies for enhancing pool boiling heat transfer using micropatterned surfaces have been conducted for the heat dissipation of very large-scale integrated circuits (VLSI) [1,2,3,4]. Wei et al. [1] obtained 4.2× enhancement in critical heat flux (CHF) using micro-fin-pin structures. Chu et al. [2] analyzed the effect of the micropatterned surface area on the CHF and concluded that the increase in capillary force in micropatterns promotes the circulation of the working fluid, thus increasing the CHF. The micropattern structures for enhanced boiling heat transfer have been commonly fabricated using a semiconductor fabrication process (e.g., photolithography) on a silicon substrate because it is a well-established fabrication method for microstructures and is compatible with integrated circuits. 

Pool boiling heat transfer has also been used in traditional heat exchange systems, such as refrigerators [5], power plants [6], and batteries [7]. For these systems, a method to fabricate a microstructure on a metallic substrate is required for achieving the enhanced pool-boiling phenomenon. Electrochemical etching is a typical method for fabricating a micropattern on a metallic substrate, in which a photolithographed or a laser-patterned barrier mask layer is used for selective etching of the metallic substrate [8,9,10]. However, the shape of the micropattern obtained by electrochemical etching is limited, owing to its isotropic etching characteristic. To fabricate engineering-designed micropatterns on a metallic substrate, researchers have proposed direct micromachining techniques, such as focused ion beam machining [11], laser machining [12], and electrochemical discharge machining [13] for metallic substrates. However, these techniques are not suitable for large-area micropatterning with a high production rate, which is important for traditional heat exchange systems. 

As an alternative method to fabricate a microstructure on a metallic substrate, a direct metal forming (DMF) method is proposed, in which a high hardness mold with microcavities is pressed against the metallic substrate at room or high temperature to transfer the micropattern onto the substrate. Tran et al. [14,15] successfully fabricated a micropattern on an aluminum (Al) alloy substrate by using a DMF process at or near the melting temperature of the substrate with a silicon micromold fabricated by a deep reactive ion etching process. Buzzi et al. [16] and Hirai et al. [17] extended the DMF process to nanoscale patterns on silver and gold substrates using silicon molds. Although silicon molds were used for DMF in previous research studies, silicon is a brittle material and, thus, is not suitable for repeated DMF processing. Nagato et al. [18] fabricated a reverse-pyramid micropattern on an Al substrate using a DMF process at room temperature with an electroformed nickel mold. Although a reverse-pyramid micropattern can be formed by a DMF process at room temperature owing to its geometrical advantage, a hot DMF process is preferred for other complex microstructures and for reducing applied pressure, which might deteriorate the micromold. Since a micromold with high hot hardness is preferred for a hot DMF process, the developments of a mold material and its patterning method are still required. 

**Figure 1:**
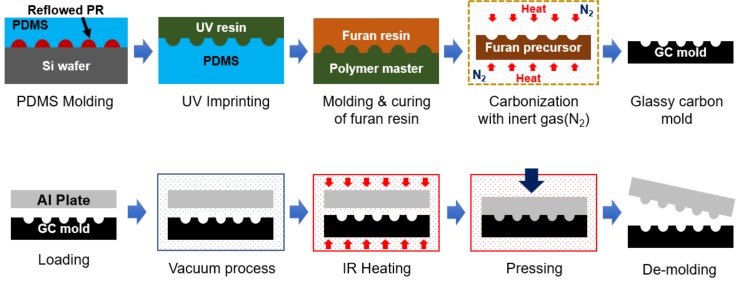
Schematic of a fabrication process for the glassy carbon (GC) mold and the direct metal forming (DMF) process.

In this study, we selected glassy carbon (GC) as a mold material for the proposed DMF process. GC (also called vitreous carbon) is a nongraphitizing carbon material [19] and shows high hot hardness and chemical/mechanical resistance [20], which are the required properties for the mold of high-temperature replication processes such as DMF and glass molding. In our previous research, we fabricated a GC micromold using carbonization of a replicated polymer precursor for glass molding application [21,22,23,24]. In this research, we applied the GC mold to the DMF of the Al substrate. Figure 1 shows the schematics of the fabrication process of the GC mold and of the DMF process with the GC mold. A microdome structure photoresist (PR) master pattern was fabricated by photolithography and a thermal reflow process, and a polydimethylsiloxane (PDMS) and UV-curable photopolymer intermediate molds were sequentially replicated from the master pattern. The furan precursor with a microdome-cavity structure was replicated from the photopolymer mold by using a thermal curing process, and the GC mold was obtained by carbonization of the furan precursor. In the DMF process, an Al substrate was placed on a GC mold and sufficient heat and pressure were applied under a vacuum environment to transfer the micropattern onto an Al substrate without oxidation. For practical application of the DMF process with a GC mold, we examined the enhanced boiling heat transfer characteristics of the fabricated DMF microdome Al substrates and we compared the CHF and the heat transfer coefficient (HTC) of the microdome structure with those of a bare Al substrate.

## 2. Fabrication the GC Mold with Macrodome Cavity

A PR master having a microdome array pattern with a pitch of 12.7 µm, a diameter of 10.6 µm, and a sag height of 1.057 µm was fabricated using conventional photolithography and a thermal reflow process [23]. A mixture of a PDMS base material and an initiator (Sylgard 184A and 184B, Dow Corning Korea Ltd., Seoul, Korea) with a mixing ratio of 10:1 was poured onto the PR master pattern and cured at room temperature for 24 h. To obtain an intermediate mold with a positive microdome pattern, we replicated a UV-curable photopolymer mold from the PDMS mold having negative microdome cavities. A UV-curable urethane acrylate photopolymer (UP088, SK Chemicals Co., Ltd., Seongnam, Korea) was poured onto the PDMS mold, and a primer-coated polyethylene terephthalate (PET) film (SH34, SKC Co., Ltd., Seoul, Korea) was used to cover it. Using a rolling process on the PET film, we moved the air bubbles trapped in the photopolymer to the outside of the sample and obtained a uniform photopolymer layer. After the UV irradiation process for 3 min, the cured photopolymer intermediate mold with a size of ~50 × 50 mm^2^ was released from the PDMS mold. 

We selected a furan resin (Kangnam Chemical Co. Ltd., Gwangju, Gyeonggi, Korea) as a precursor material for the GC mold owing to its high carbon yield. A furan resin mixture composed of 89.8 wt.% furan resin, 0.2 wt.% *p*-toluenesulfonic acid (CH_3_C_6_H_4_SO_3_H H_2_O, PTSA; Kanto Chemical Co. Inc., Tokyo, Japan), and 10 wt.% ethanol was prepared, and a degassing process was conducted for 2 h in a vacuum chamber to remove the entrapped air bubbles in the furan mixture. The degassed furan mixture was poured onto the photopolymer intermediate mold, and a two-step thermal curing process was carried out to avoid warpage of the replicated furan precursor [24]. The furan mixture was first cured under an atmospheric condition for 5 days and then cured again in a convection oven at a maximum temperature of 100 °C. In the second curing process, the rate of temperature increase was set to 0.1 °C/min, and the temperature was maintained for 60 min for every 5 °C temperature increment until the maximum temperature was reached. Finally, the microdome cavity arrayed furan precursor was released from the photopolymer mold and the backside of the furan precursor was polished to obtain the desired thickness and flatness. 

**Figure 2:**
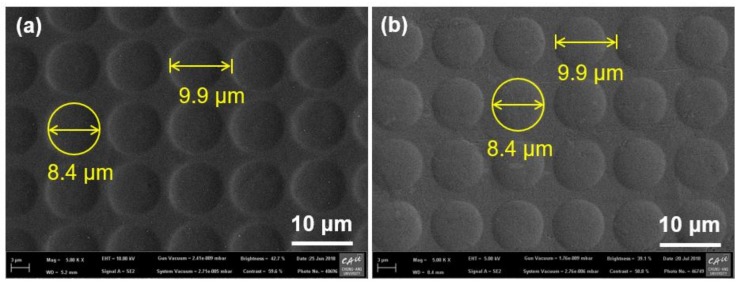
Scanning electron microscope (SEM) images of (**a**) microdome cavities on GC mold and (**b**) microdome array on aluminum (Al) substrate fabricated by DMF with a temperature of 645 °C and a pressure of 2 MPa.

To obtain the GC mold, we carried out carbonization of the fabricated furan precursor in a tube furnace under a vacuum environment (modified MIR-TB1001-2; Mirfurnace Co. Ltd., Pocheon, Korea). To allow the slow escape of pyrolysis gases (i.e., H_2_, CH*_x_*, CO_2_, and CO) from the GC mold and avoid warpage of the GC mold during the carbonization, we gradually increased the temperature of the furnace at a rate of 0.5 °C/min until it reached 600 °C. Beyond 600 °C, the temperature was increased at a rate of 1 °C/min until it reached the maximum temperature of 1000 °C and then maintained at this level for 10 h. After the carbonization, a GC mold with microdome cavities was obtained. Figure 2a shows a scanning electron microscopy (SEM) image of the fabricated microdome cavities on the GC mold. The measured pitch, diameter, and height of the microdome cavity array were 9.9 µm, 8.4 µm, and ~0.76 µm, respectively. The differences in dimensional properties between the PR master and the GC mold were mainly due to the material decomposition in the carbonization process. The shrinkage ratio (reduction ratio of the measured dimensions of the GC mold to the PR master) for the pitch and diameter (inplane) was ~21.4% and that for the height (out-of-plane) was ~28%. Although an anisotropic shrinkage was observed, this shrinkage can be predicted and compensated.

## 3. Fabrication of a Microdome Patterned Al Substrate by DMF

**Figure 3:**
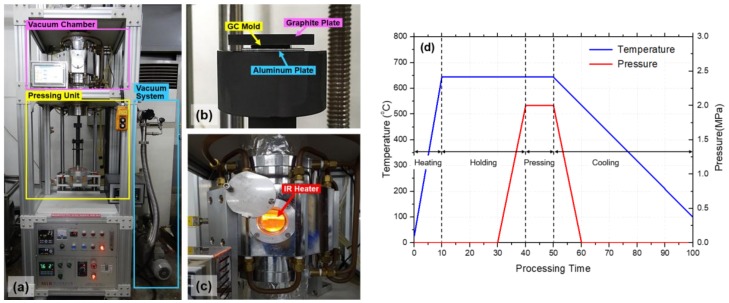
(**a**–**c**) Photographs of constructed DMF system; (**a**) whole system; (**b**) graphite pressing jig unit with Al substrate, GC mold, and cover graphite plate; and (**c**) vacuum chamber with infrared heater during the heating stage; (**d**) pressure and temperature histories in DMF process.

A DMF system consisting of an infrared (IR) heater for heating up to 1050 °C with a heating rate of 70 °C/min, a motorized pressing unit with a maximum compression force of 150 kgf, and a vacuum chamber was designed and constructed, as shown in Figure 3a. Figure 3b shows the graphite pressing jig unit with an Al substrate (AA1050), a GC mold, and a cover graphite plate. The cover graphite plate was used for applying a preload to prevent any slight movement of the GC mold and Al substrate during the following evacuation process. At the starting point of the DMF process, the pressing jig unit with samples moved up into the vacuum chamber with an IR heater. After the evacuation process, to prevent oxidation, the environmental temperature of the vacuum chamber was increased by the IR heater, as shown in Figure 3c. The DMF process was divided into four stages: heating, holding, pressing, and cooling. Figure 3d illustrates the pressure and temperature history during the DMF process. In the heating stage, the ambient temperature of the GC mold and Al substrate was increased up to the forming temperature of 645 °C. In the holding stage, the temperature was maintained for 30 min to achieve a uniform temperature distribution of the GC mold and Al substrate. In the pressing stage, a compression pressure of 2 MPa was applied to the stack of the GC mold and Al substrate for 10 min. In the cooling stage, the pressure was released and the IR heater was turned off for natural cooling. When the furnace temperature was reduced to room temperature, the pressing jig unit was lowered down from the vacuum chamber and the formed Al substrate was detached from the GC mold.

**Figure 4:**
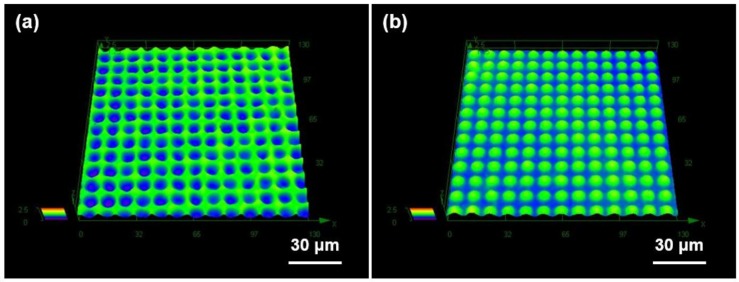
Three-dimensional surface profiles of (**a**) a GC mold and (**b**) a DMF microdome Al substrate obtained by laser confocal microscope.

In the DMF process, the processing temperature and pressure are the important parameters affecting the quality of the metallic microstructure. The ductility of the Al substrate is not enough to lead to a perfect plastic deformation at a low temperature, and the Al substrate can be liquefied at or near the melting temperature. Although higher compression pressure is preferred for improving the replication quality of the metallic microstructure, excessive pressure might deteriorate the GC mold. To determine the appropriate processing condition of the DMF process, we conducted multiple DMF experiments using an Al substrate with a thickness of 1 mm by increasing the processing temperature with a fixed compression pressure of 2 MPa, which was the maximum compression pressure limit to avoid damage to the fabricated GC mold. On the basis of the repeated DMF experiments with various processing temperatures, we selected a processing temperature of 645 °C, which provided sufficient DMF quality. Figure 2b shows the uniformly distributed microdome-structured Al substrate produced by the DMF process at a processing temperature of 645 °C and a compression pressure of 2 MPa. The measured diameter and pitch of the microdome array on the Al substrate obtained from the SEM image were exactly the same as those of the GC mold. Figure 4 shows the three-dimensional surface profiles of (a) the GC mold and (b) the fabricated DMF microdome Al substrate obtained by a laser confocal microscope (OLS-4100, Olympus. Co. Ltd., Tokyo, Japan). The measured height of the microdome on the GC mold was ~0.76 µm and that on the Al substrate was ~0.74 µm. The differences between the GC mold and the DMF microdome Al substrates were negligible, considering the measurement errors in the positive and negative shapes using an optical height measurement system.

The thickness of the Al substrate could affect the replication quality of the DMF process. Figure S1 in the supplementary material shows the effect of the Al substrate thickness (0.16, 0.2, 0.3, and 1.0 mm) on the height of the fabricated microdome. It was noted that the effects of Al substrate thickness were negligible when the substrate thickness was thicker than 0.16 mm. It might be because the height of the microdome was much smaller than the thickness of Al substrate.

Since the DMF process was conducted at a high temperature, some chemical reactions including element diffusion could have occurred during the process. To examine the chemical reaction during the DMF process, we analyzed the element compositions of the GC mold and the Al substrate before and after the DMF process using energy-dispersive X-ray spectroscopy (EDX), as shown in Figure S2 (supplementary material). It was noted that there were no significant changes in the element compositions of both the GC mold and the Al substrate during the DMF process owing to the high chemical resistance of the GC mold. 

## 4. Application of the DMF Microdome Al Substrate to Enhanced Boiling Heat Transfer 

### 4.1. Experimental Setup and Measuring Method for Boiling Heat Transfer

**Figure 5:**
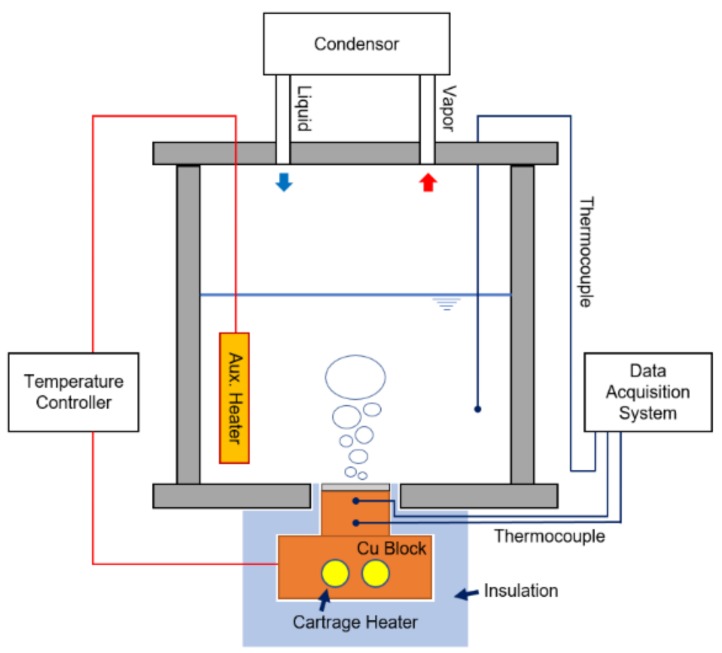
Schematic of experimental setup for boiling heat transfer using fabricated sample.

As a practical application of the DMF microdome Al substrate, the enhanced boiling heat transfer characteristic of the fabricated sample was examined. Figure 5 shows the schematics of the experimental setup and the analysis system for pool boiling heat transfer. The liquid chamber with a volume of 3.3 L was made of stainless steel (SUS 330L) to prevent corrosion and reaction with other materials. As a heating source, a copper block (thermal conductivity = 401 W/(m·K)) was precisely machined and two cartridge heaters (totaling 600 W maximum) were inserted in the bottom of the block. Moreover, a set of auxiliary heaters was installed inside of the liquid chamber. The power of the cartridge heaters and of the auxiliary heaters was controlled by a proportional-integral-derivative (PID) controller. The sample was attached to the copper block using a thermal grease (MX-4, ARCTIC (HK) Ltd., Hong Kong, China; thermal conductivity = 4.01 W/(m·K)). Finally, the copper block was attached on the bottom of the liquid chamber. Every interface was sealed with a Teflon gasket to prevent any leakage of the boiling liquid. At the top of the liquid chamber, an air-cooled condenser was installed to liquefy the vapor. The outside of the copper block was insulated with silicone (thermal conductivity = 0.2 W/(m·K)) and Teflon (thermal conductivity = 0.25 W/(m·K)) sheets. In this setup, the copper block had a higher thermal conductivity than that of the insulation materials. Therefore, it could be assumed that the heat transfer on the copper block was one dimensional because the heat loss of the side wall was very small and, thus, negligible [25]. Two small holes were drilled in the middle of the copper block, and a couple of thermocouples (J-Type, Omega Engineering Inc., Stamford, CT, USA) were inserted into the holes to calculate the heat flux on the sample surface. The measured data were collected by a multichannel data logger system (Graphtec GL220, DATAQ Instruments Inc., Akron, OH, USA). As a working fluid, the FC-72 coolant (3M Inc., Maplewood, MN, USA) was used. To calculate the heat flux *q″*, we applied Fourier’s law, which is described in Equation (1):

(1) $$q^{\prime \prime }=-k\frac{dT}{dz}=-k_{Cu}\frac{T_{H}-T_{L}}{d_{a}}\;$$

where the temperature gradient (d*T*/d*z*) was estimated by measuring the temperature difference (*T_H_* − *T_L_*) and the axial distance *d_a_* between the two thermocouples, as shown in the right-hand side of Equation (1). The temperature of the sample surface *T_s_* can be calculated using the heat flux and the lower temperature measured from the thermocouple *T_L_* from the following equation:

(2) $$T_{S}=T_{L}-q^{\prime \prime }\left(\frac{L_{Cu}}{k_{Cu}}+R_{tc}+\frac{L_{Al}}{k_{Al}}\right)\;$$

where the contact resistance *R_tc_* was calculated as 0.095 cm^2^·K/W by utilizing the procedure reported by Cooke et al. [26]. Moreover, wall superheat Δ*T_S_* was defined as the temperature difference between the surface temperature *Ts* and the saturation temperature of the boiling liquid *T_sat_* (FC-72, 56 °C):

(3) $$\Delta T_{S}=T_{S}-T_{Sat}=T_{S}-56\;{}^{\circ}C\;$$

Finally, the HTC *h_S_* was calculated using Newton's law of cooling:

(4) $$h_{S}=\frac{q^{\prime \prime }}{T_{S}-T_{Sat}}=\frac{q^{\prime \prime }}{\Delta T_{S}}\;$$

### 4.2. Uncertainty Analysis 

In the boiling heat transfer experiment, uncertainties existed owing to the errors in the machining of temperature measurement points, errors in the thermocouples for measuring the temperature, and variations in the thermal conductivity of the materials. The positioning accuracy of the holes for temperature measurement was ±0.01 mm, and the measurement error of the J-Type thermocouple was ±0.15 K. The variation in the thermal conductivity with the temperature change was ±2% for copper and ±2.1% for Al, and the uncertainty of the contact resistance *R_tc_* was ±2.37%. The overall uncertainty was calculated using the second-power equation proposed by Kline et al. [27]. As a result, the calculated uncertainties of the heat flux and the HTC were 8.72% and 8.74%, respectively. Table 1 summarizes the uncertainty of the pool boiling heat transfer experiment and the calculated values.

### 4.3. Experimental Result and Discussion

A series of boiling experiments was conducted to evaluate the heat transfer performance of the DMF microdome Al substrates. For the comparison, a bare Al substrate was also evaluated using the same experimental procedure. For each set of the boiling experiments, boiling liquid was heated above the saturation temperature (FC-72, 56 °C) for 10 min to eliminate the gases trapped or dissolved in the boiling liquid because they could affect the heat transfer performance in the pool boiling case. After the degasification process, the system was left for an hour at the saturation temperature until it reaches a thermally stable state. Then, the temperature of the copper block was controlled in increments of 1 °C for every minute until the onset of boiling had occurred. Once the boiling had started, the heat flux was increased continuously. From this point, the temperature increment of the copper block was adjusted to 0.5 °C for every minute.

**Figure 6:**
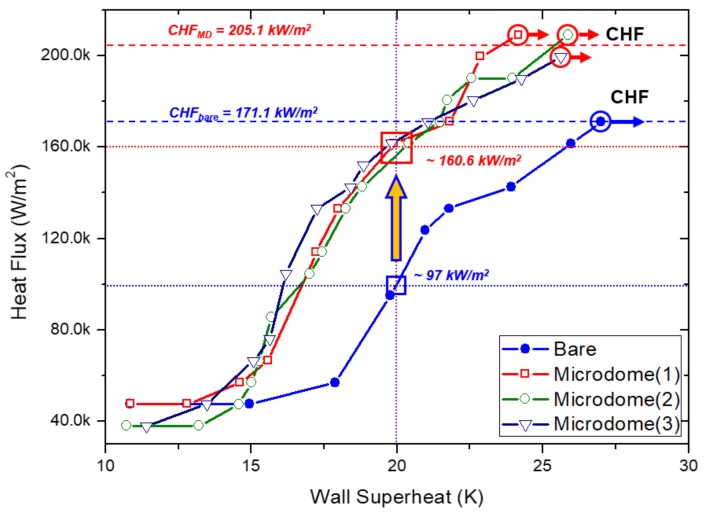
Comparison of the boiling curves of three DMF microdome Al substrates and a bare Al substrate.

Figure 6 shows the comparison result of the boiling curves for the three DMF microdome Al substrates fabricated at the optimum condition and for the bare Al substrate. In the case of the bare Al substrate, the onset of boiling was started at ~15 °C of the wall superheat temperature. As the wall superheat temperature increased, the heat flux simultaneously increased until the wall superheat temperature reached ~27 °C. As shown in Figure 6, at this point the heat flux was calculated as 171.1 kW/m^2^ as a CHF. Above the CHF point, film boiling occurred and the wall superheat temperature increased rapidly within a short time. Thus, the experiment was terminated when film boiling had occurred. In the case of the DMF microdome Al substrates, the onset of boiling was started at ~14 °C of the wall superheat temperature. It shows that the microdome structure can reduce the overheating temperature leading to the boiling. In the same manner as with the bare Al substrate, boiling experiments were performed until film boiling had occurred. The average value of the CHF for the three DMF microdome Al substrates was calculated as 205.9 kW/m^2^ with ± 4.5 kW/m^2^ of standard deviation, when the wall superheat temperature reached ~25 °C. This value was 20.4 ± 2.6% higher than that for the bare Al substrate. At the wall superheat temperature of 20 °C, the calculated heat flux values of the three DMF microdome Al substrates and the bare Al substrate were ~160.6 ± 2.7 kW/m^2^ and ~97 kW/m^2^, respectively. It shows that the microdome structure enhanced the heat flux by 65.6 ± 2.8% in the moderate boiling regime.

HTC is another important parameter for evaluating the performance of pool boiling heat transfer. The HTC value (*h_c_*) was calculated using Equation (4). The maximum HTC values of the bare Al substrate and the DMF microdome Al substrates were calculated as 5.96 kW/(m^2^·K) and 8.0 ± 0.3 kW/(m^2^·K), respectively. Because of the definition of Newton’s law of cooling, the HTC is proportional to the heat flux and inversely proportional to the wall superheat temperature. In the case of the bare Al substrate, the overall heat flux was lower and the wall superheat temperature was higher than those of the DMF microdome Al substrates. This explains why the HTC value of the DMF microdome Al substrates was 34.1 ± 5.3% higher than that of the bare Al substrate. 

## 5. Conclusions

In this paper, we propose a DMF process using a GC mold. The GC mold was prepared by carbonization of a replicated furan precursor, and a standalone-type DMF system was designed and constructed. To examine the feasibility of the proposed method, we successfully fabricated an array of microdome structure with a pitch of 9.9 µm, a diameter of 8.7 µm, and a height of ~0.74 µm on an Al substrate by using DMF with a processing temperature of 645 °C and a compression pressure of 2 MPa. For a practical application, we examined the enhanced boiling heat transfer characteristics of the DMF microdome Al substrates and compared them with those of the bare Al substrate. The DMF microdome Al substrates showed 20.4 ± 2.6% higher CHF and 34.1 ± 5.3% higher HTC than those of the bare Al substrate. Although we successfully demonstrated the fabrication of microdome structure on an Al substrate by DMF with GC mold for enhanced boiling heat transfer, the structural optimization is still required for maximizing CHF and HTC.

The size of the DMF microdome Al substrates (~20 × 20 mm^2^) and the standalone-type DMF system used in this study are not acceptable for a large-area micropatterning process of metallic substrates with a high production rate. However, the proposed DMF with GC mold can be extended to mass production of a large-area micropattering process of metallic substrate because multiple large-area GC molds can be obtained by the proposed GC mold fabrication process and a batch process concept using multiple molds can be applied to the DMF process. In addition, the proposed method can be extended to the roll-to-roll DMF process, which can provide high patterning speed on a large area because a GC roll mold with microcavities can be obtained by carbonization of the roll-shaped polymer precursor. The optimization of the structural parameter to maximize the boiling heat transfer performance and the development of the roll-to-roll DFM process with a GC roll mold are the subjects of our ongoing research.

## Figures and Tables

**Table 1:** Uncertainty sources and error values.

Uncertainty Source	Error
Machining error for measuring position	±0.01 mm
J-type thermocouple reading	±0.15 K
Thermal conductivity of Cu	±2%
Thermal conductivity of Al	±2.1%
Thermal contact resistance	±2.37%
Surface temperature reading	±0.62%
Heat flux	±8.72%
Heat transfer coefficient	±8.74%

## Supplementary Materials

The following are available online at http://www.mdpi.com/2072-666X/9/8/376/s1: Figure S1: Effects of the Al substrate thickness (0.16, 0.2, 0.3, and 1.0 mm) on the height of the DMF microdome structure.; Figure S2: EDX analysis results for (a) the GC mold before DMF process, (b) the GC mold after DMF process, (c) a bare Al substrate, and (d) the DMF microdome Al substrate.
