# Supplementary material for: Direct Metal Forming of a Microdome Structure with a Glassy Carbon Mold for Enhanced Boiling Heat Transfer
Source: Micromachines (Basel). 2018 Jul 28;9(8):376. doi: 10.3390/mi9080376 (PMC6187845; doi:10.3390/mi9080376)
Supplement: Supplementary file 1 [file micromachines-09-00376-s001.docx]

Supplementary Material: Direct metal forming of microdome structure with glassy carbon mold for enhanced boiling heat transfer

Jun Kim^1^, Dongin Hong^1^, Mohsin Ali Badshah^2^, Xun Lu^2^, Young Kyu Kim^2^ and Seok-min Kim^1,2,*^

^1^ Department of Mechanical System Engineering, Chung-Ang University, Heukseok-dong, Dongjak-gu, Seoul 06974, Republic of Korea; zuhn@cau.ac.kr (J.K), hdi2305@naver.com (D.H)

^2^ Department of Mechanical Engineering, Chung-Ang University, Heukseok-dong, Dongjak-gu, Seoul 06974, Republic of Korea; mohsinali@cau.ac.kr (M.A.B), luxun@cau.ac.kr (X.L), kykdes@cau.ac.kr (Y.K.K)

***** Correspondence: smkim@cau.ac.kr; Tel.: +82-2-820-5877

A1. The effect of thickness of the Al substrates on the DMF process

The thickness of the Al substrates can affect the replication quality of the DMF process. Thus, we carried out some DMF processes with Al substrates with different thicknesses (0.16, 0.2, 0.3, and 1.0 mm) at a processing temperature of 645 °C and a pressure of 2 MPa. Figure S1 shows the effects of the Al substrate thickness on the height of the DMF-fabricated microdome structure. We measured the height of the randomly selected 10 microdomes for each DMF-fabricated Al substrate. It shows that the averaging values of the height of microdomes were almost same as ~0.74 um. Thus, the effects of the Al substrate thickness were negligible when the Al thickness was thicker than 0.16 mm.


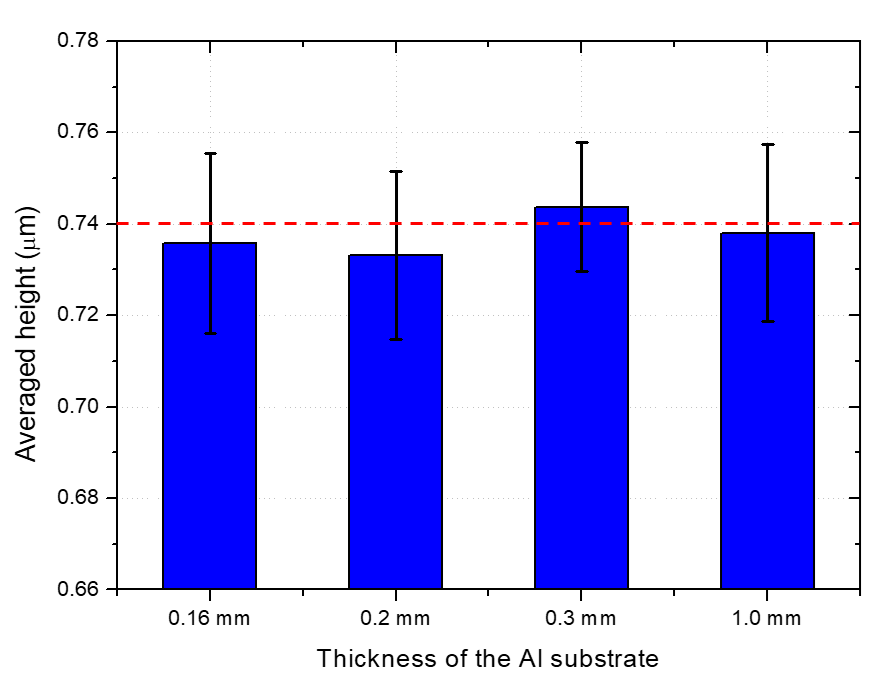


Figure S1 Effects of the Al substrate thickness (0.16, 0.2, 0.3, and 1.0 mm) on the height of the DMF microdome structure.

A2. Reaction between the glassy carbon (GC) mold and the aluminum (Al) substrate during the direct metal forming (DMF) process

To examine the chemical reaction or element diffusion during the DMF process, we analyzed the element compositions of the GC mold and the Al substrate before and after the DMF process using energy-dispersive X-ray spectroscopy (EDX). A newly fabricated GC mold was used for the measurement of the before-DMF sample, and a GC mold after more than 10 times DMF processes with Al substrates was used for the measurement of the after-DMF sample. Figure S2 shows EDX analysis results for (a) the GC mold before DMF process, (b) the GC mold after DMF process, (c) the bare Al substrate, and (d) the DMF microdome Al substrate. It clearly shows there were no significant element composition changes during the DMF process. Table S1 shows the summary of the element compositions for each sample measured by EDX. For the GC mold, a small amount of Mg atom was newly detected on the GC mold after DMF process. However, it can be negligible because the Mg atom was not detected on the Al substrate and the amount of Mg atom is much smaller than that of C atom. For the Al substrate, the amount of O atom was increased during the DMF process because of a little oxidization of Al substrate during the DMF process. However, the increase in the amount of C atom was not observed on DMF Al substrate. These results clearly show that there was no chemical reaction or element diffusion during the DMF process using GC mold.


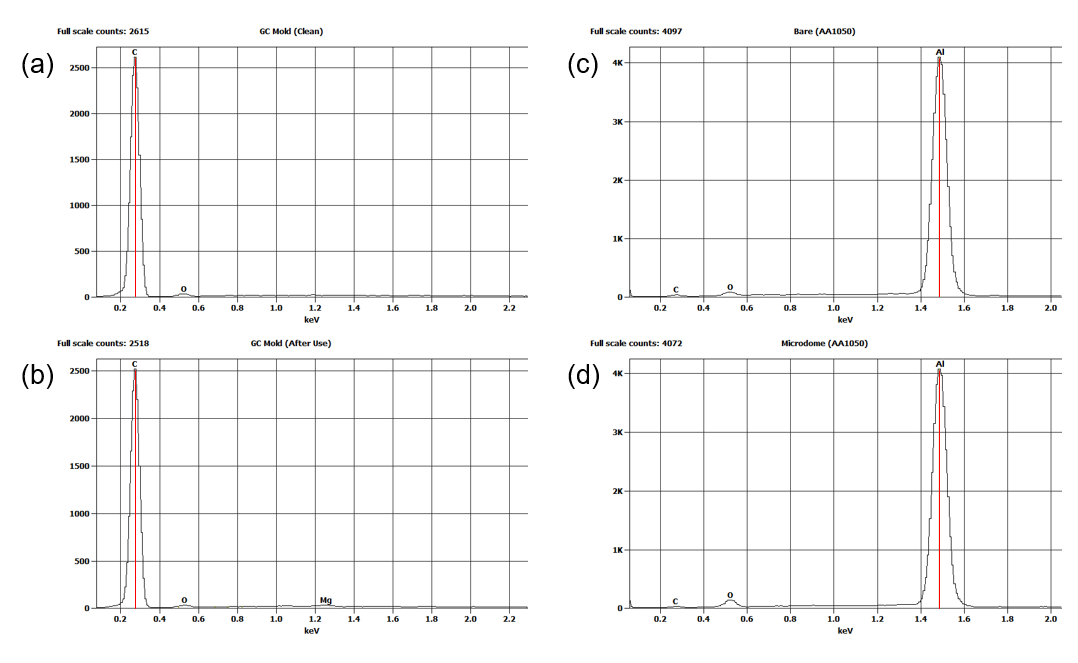


Figure S2 EDX analysis results for (a) the GC mold before DMF process, (b) the GC mold after DMF process, (c) a bare Al substrate, and (d) the DMF microdome Al substrate.

**Table S1.** Summary of the element compositions for each sample measured by EDX.

|  | GC mold (before) | | GC mold (after) | | bare Al substrate | | DMF Al substrate | |
| --- | --- | --- | --- | --- | --- | --- | --- | --- |
|  | wt. % | atom % | wt. % | atom % | wt. % | atom % | wt. % | atom % |
| C | 97.46 | 98.08 | 96.57 | 97.78 | 1.95 | 4.22 | 1.52 | 3.25 |
| O | 2.54 | 1.92 | 1.93 | 1.46 | 2.44 | 3.95 | 5.14 | 8.22 |
| Mg | - | - | 1.50 | 0.75 | - | - | - | - |
| Al | - | - | - | - | 95.61 | 91.84 | 93.33 | 88.53 |
| Total | 100 % | | | | | | | |
